# Bonding Behavior of Conventional PMMA towards Industrial CAD/CAM PMMA and Artificial Resin Teeth for Complete Denture Manufacturing in a Digital Workflow

**DOI:** 10.3390/ma14143822

**Published:** 2021-07-08

**Authors:** Dominik Klaiber, Sebastian Spintzyk, Juergen Geis-Gerstorfer, Andrea Klink, Alexey Unkovskiy, Fabian Huettig

**Affiliations:** 1 Section “Medical Materials Science and Technology”, University Hospital Tuebingen, 72076 Tübingen, Germany; dominik-klaiber@web.de (D.K.); sebastian.spintzyk@med.uni-tuebingen.de (S.S.); juergen.geis-gerstorfer@med.uni-tuebingen.de (J.G.-G.); 2 Department of Prosthodontics, Center for Dentistry, Oral Medicine and Maxillofacial Surgery, University Hospital Tuebingen, 72076 Tübingen, Germany; andrea.klink@med.uni-tuebingen.de; 3 Department of Prosthodontics, Geriatric Dentistry and Craniomandibular Disorders, Charité—Universitätsmedizin Berlin, Corporate Member of Freie Universität Berlin, Humboldt-Universität zu Berlin, 14197 Berlin, Germany; alexey.unkovskiy@charite.de; 4 Department of Dental Surgery, Sechenov First Moscow State Medical University, 119991 Moscow, Russia

**Keywords:** dental prosthesis, denture bases, prosthodontics, edentulous jaw, self-curing of dental resins

## Abstract

When applying a digital workflow, custom artificial resin teeth have to be integrated into a milled complete denture base, using polymethylmethacrylate (PMMA) applied with a powder–liquid technique. Debonding of denture teeth from dentures is reported to be a frequent complication. No evidence is provided as to which method of surface treatment may enhance the bonding strength. The bonding strength between artificial teeth and PMMA (Group A, *n* = 60), as well as between the PMMA and industrial PMMA (Group B, *n* = 60), was investigated following no treatment, monomer application, sandblasting, oxygen plasma, and nitrogen plasma treatment. Surface-roughness values and SEM images were obtained for each group. Shear bond strength (SBS) and fracture mode were analyzed after thermocycling. Within Group A, statistically significant higher SBS was found for all surface treatments, except for nitrogen plasma. In Group B, only nitrogen plasma showed a statistically lower SBS compared to the reference group which was equivalent to all surface treatments. Conclusions: Within the limitations of the present study, the monomer application can be proposed as the most effective surface-treatment method to bond custom artificial teeth into a milled PMMA denture base, whereas nitrogen plasma impairs the bonding strength.

## 1. Introduction

Despite of great strides in prophylaxis and a higher number of remaining teeth in the elderly by 2030 in Germany, the amount of patients in need of complete dentures due to edentulism will keep prevalent in industrialized countries, e.g., with about 1 to 2 million people in Germany during the upcoming decade [1,2]. Furthermore, complete tooth loss is a major burden of disease worldwide [3].

A conventional workflow to fabricate a complete denture implies manual and time-consuming waxing of the base with prefabricated artificial resin teeth, and its transfer into polymerized acrylate. Commonly, denture bases are made from polymethylmethacrylate (PMMA) based on a powder-liquid system, whereas denture teeth are mostly made from micro-filled reinforced polyacrylic or a micro-/nano-filled composite material.

The recent advancements in computer-aided design and manufacturing (CAD/CAM) offer a digitalization of the whole production chain for complete dentures [4,5,6,7]. The milling of a denture base using prefabricated acrylic resin blanks was reported to have some clinical advantages over the conventional workflow [8]. Besides aspects in the lab workflow, some industrial polymerized PMMA blanks offer a lower monomer release over time [9,10], but all offer an initially low monomer release. This leads to the same problem of inferior bonding capacity as known from industrial custom resin teeth (also made from PMMA) [11].

In this “conventional workflow”, the bonding between denture base and denture teeth occurs during the polymerization of PMMA in presence of the artificial resin teeth. In a “digital workflow”, the bonding of both polymerized entities (teeth and base) has to be performed separately. This calls for a surface conditioning, a bonding material, or the simple application of polymerizing PMMA (powder-and-liquid technique) [12]. The debonding of denture teeth from the denture base is the most reported complication in full and partial dentures [13,14,15]. Debonding is mostly caused by an insufficient connection due to impurities or decontaminations of the corresponding surfaces, especially wax remnants from the conventional workflow [14,16,17].

Thus, numerous studies focused on the enhancement of bonding strength between denture bases and denture teeth. In example, Barbosa et al. showed an improvement of bonding strength after wax solvent and monomer (methacrylate) application [18,19]. Furthermore, other surface conditioning methods, such as YAG laser irradiation [20,21], plasma pretreatment [22,23], may be applied to enhance the bonding strength of acrylic resin teeth to denture bases, with airborne-particle abrasion and sandblasting being mostly used [20,21].

However, the topical literature lacks evidence, which of the mentioned pretreatments improves the bonding strength when bonding artificial teeth with PMMA to PMMA.

For this reason, the aim of the present study was to quantify the influence of monomer application, sandblasting, and cold-plasma treatment on the bonding strength between milled PMMA denture bases and custom artificial resin teeth.

The two null hypotheses of this study were that the applied surface-treatment methods would make no difference in bond strength between PMMA denture base and artificial teeth when bonded with polymerizing PMMA.

## 2. Materials and Methods

**Figure 1:**
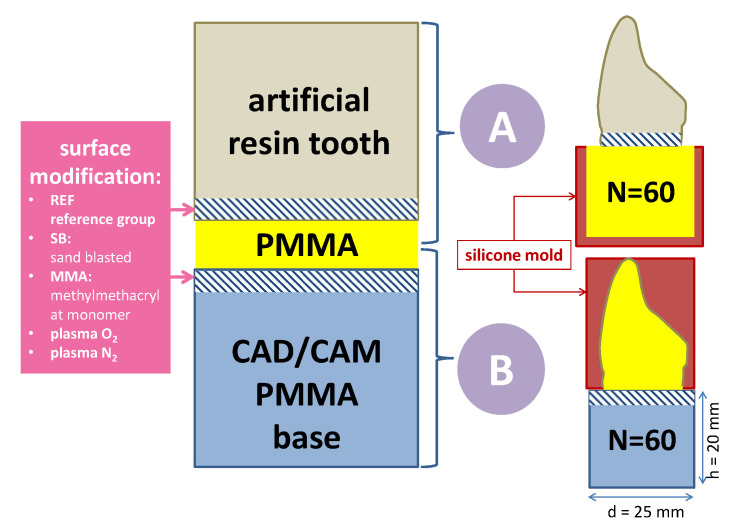
Schematic drawing of the interface and experimental conditioned surfaces towards the bonding interface by polymerizing PMMA (powder + liquid system). Consequently, two groups of specimens (*n* = 60 per group) are subdivided per surface modification (*n* = 12 per subgroup).

To compare the bonding efficiency of experimental conditionings applied to artificial resin teeth and CAD/CAM fabricated PMMA denture base, two study groups were set: Group A for evaluation of the bond strength between teeth and a polymerizing PMMA fixation layer. This Group A can be regarded as a group of bonding comparable to the conventional approach. Group B addresses the bonding between the denture base and the polymerizing PMMA fixation layer. Therewith it characterizes the bonding interface to the industrial polymerized PMMA. The bonding interface of the tooth (Group A) and of the denture base specimen (Group B) has to be modified prior to mating of the interface with polymerizing PMMA in powder-liquid techniques. Thus, there were two kinds of specimens (Figure 1). Further material specifications can be found in Table A1, Appendix A. 

### 2.1. Arrangements for the Specimens in Group A (Conventional PMMA to Resin Tooth)

For Group A, sixty artificial teeth (*n* = 120; Mondial 6 R483, Kulzer, Hanau, Germany) were grinded at the basal surface (HSS99-KFO, Wassermann, Hamburg, Germany) to standardize the bonding area. The surfaces were measured by using stereomicroscopy (M400, Wild Heerbrugg, Switzerland) and supported by software (Measure, Datinf, Tuebingen, Germany) to evaluate the bonding surface “A” for further calculations. A cylindrical silicone mold (25 mm in diameter × 20 mm height) was made on the basis of a CAM-milled PMMA cylinder used in Group B.

### 2.2. Arrangements for the Specimens in Group B (CAD/CAM PMMA to Conventional PMMA)

**Figure 2:**
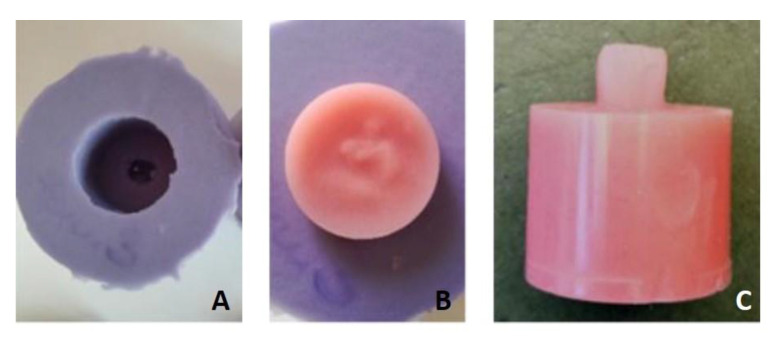
Replication of the tooth shape for Group B (CAD/CAM), as used in Group A: (**A**) silicone mold for duplication of the artificial tooth shape; (**B**) auto-curing PMMA being applied into the silicone mold in liquid state—the milled PMMA block with prepared surface conditioning is put on top of it in order to mate it; (**C**) the finalized shear bond testing specimen of Group B.

For Group B, the sixty cylindrical test blocks (25 mm × 20 mm) were designed in CAD software (Autodesk Fusion 360, San Rafael, CA, USA) (STL provided as Supplementary File S1) and milled from prefabricated PMMA blanks (pink blank, Anaxdent, Stuttgart, Germany) by using a CNC machine (550i, imes-icore, Eiterfeld, Germany). A mold was made from a silicone material (Dublisil 30, DreveDentamid GmbH, Unna, Germany) to replicate the shape of the artificial resin tooth as used in Group A (Figure 2).

### 2.3. Surface Conditioning, Mating, and Ageing of Specimens

**Figure 3:**
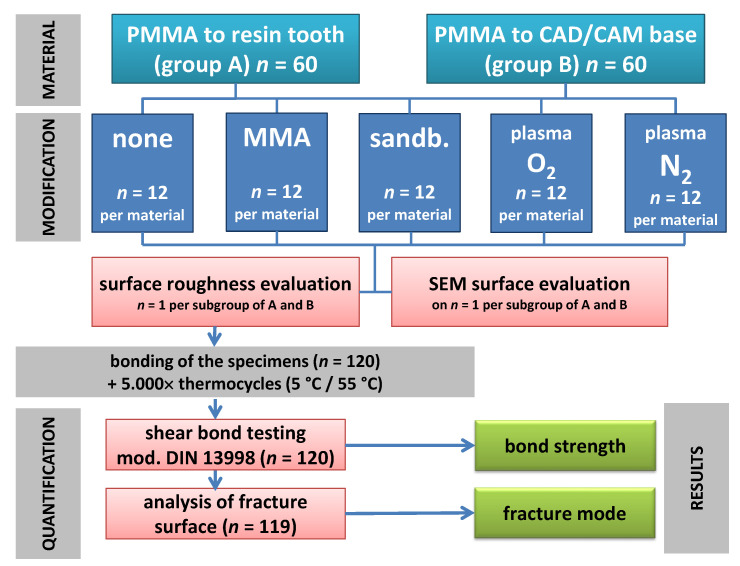
Overview of the study measures in both groups (A and B) resulting in 5 subgroups by surface treatment prior to mating.

The 120 specimens of Groups A and B were divided in five subgroups according to the five surface conditionings (Figure 3). In the reference group, the specimens were left untreated (as is). In the MMA group, the MMA-monomer (megaCRYL, Megadental, Büdingen, Germany) was applied on the specimens’ surface with microbrushes for 30 s and left to dry. In the plasma groups the specimens were pretreated with 100% O_2_ and 100% N_2_ feeding gases for 30 min, respectively, by 0.3 mbar and 60% power.

In the sandblasting group, the specimens were treated with a 250 µm Al_2_O_3_ (Korox 250, Bego GmbH, Bremen, Germany) at 0.3 MPa for 30 s in 5 mm distance (P-G 400, Harnisch-Rieth GmbH & Co., Winterbach, Germany) and cleaned by water steam for 10 s.

After conditioning of the artificial resin teeth in Group A, the PMMA liquid and powder (megaCRYL, Megadental, Büdingen, Germany) were mixed according to manufactures instructions and poured into the cylindrical silicone mold to polymerize in a pressure pot, under 0.25 MPa, in 40 °C water, for 25 min (as recommended by the manufacturer).

After conditioning of the CAD/CAM PMMA in Group B, the tooth-shaped silicon mold was poured with mixed powder-liquid PMMA and set on the modified surface of the cylinder (. Thereafter, it was cured under the same conditions as mentioned above. All resin residuals were removed with a cross-edged cutter.

All specimens (Groups A and B) were artificially aged by 5000 thermocycles (5 °C; 55 °C; 30 s dwelling time) with a standard testing machine (Haake W15, SD Mechatronik, Feldkirchen-Westerham, Germany).

### 2.4. Surface Characterization

After surface treatment of each subgroup three specimens underwent a topographic analysis towards the average surface roughness (Ra) measured with a contact profilometer (Perthometer S6P, Mahr, Göttingen, Germany).

Therefore, an area of 3 mm × 3 mm was examined with 121 orthogonal measurements. The measured topography was further analyzed by using software (Mountains Map V7.3, Digital Surf, Besançon, France) with a Gaussian filter of 0.6 mm.

One specimen per group was analyzed via scanning electron microscopy (SEM) (LEO 1430, Zeiss, Oberkochen, Germany) in 500× and 5000× magnification in order to determine the failure mode after the bond test.

### 2.5. Assessment of the Bonding Strength and Failure Mode

A universal testing machine (Z010, Zwick, Ulm, Germany) running with a crosshead speed of 1 mm/min towards the specimen mounted in a fixation device with a 45° position to the piston was used to determine the fracture load (“F”, in Newton). All specimens were subjected to this testing.

The bonding surface “A” was determined by averaging repeated measurements of the standardized cross-section of the artificial teeth with a value of 48.97 mm^2^.

The shear bond strength (SBS, in MPa) was calculated using the Equation (1).

(1) $$\mathrm{SBS}=\frac{F}{A}$$

Equation (1): Calculation of the shear bond strength (SBS) from the maximum load at fracture (F) in N divided by the bonding surface (A) in mm^2^. 

The failure mode of each specimen was divided into the following groups: adhesive mode, cohesive mode, and mixed mode.

### 2.6. Statistical Analysis

The statistical analysis was performed in the JMP Version 14.2 (SAS institute, Cary, NC, USA. The Ra data of the 121 measurements per specimen were analyzed for variability.

The gathered SBS data were analyzed for the normality of distribution applying Shapiro-Wilk W-test. A one-way ANOVA and post hoc Tukey tests were performed on α level 0.05 for normally distributed data.

The mean Ra values of the bonding surfaces were correlated with the SBS values of each specimen per group to identify a potential coherence of interface roughness and SBS.

## 3. Results

### 3.1. Initial Surface Characteristics

**Figure 4:**
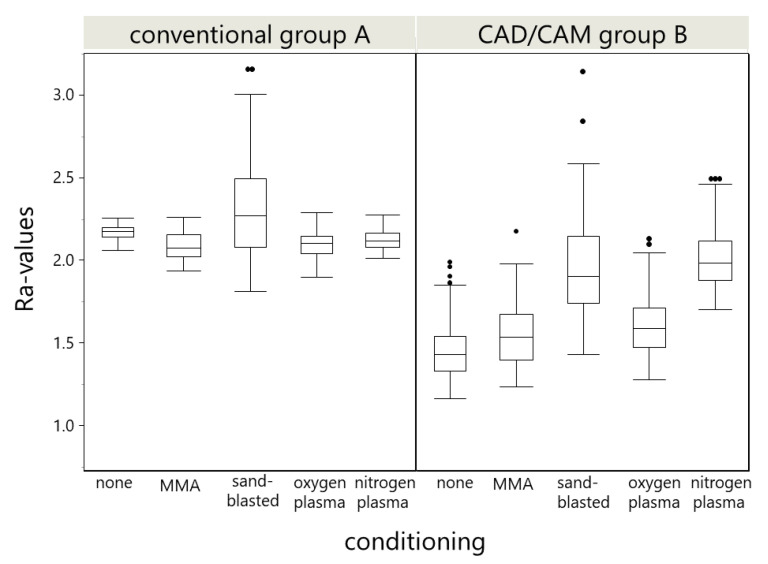
Boxplots of the Ra values (µm) of the surface modifications. Data are based on one specimen per group derived from 121 profiles every 25 µm over a 9 mm^2^ in the center of the specimen. There was no statistical significance between the Ra values of oxygen and MMA conditioning within Group A. All Ra distributions were statistically significantly (*p* < 0.01) different from each other within Group B. Furthermore, the comparison of the conditioning revealed a statistical difference (*p* < 0.0001) between Groups A and B.

The surface evaluation of the three specimens out of each material revealed differences within the Ra values given in Table 1 and depicted in Figure 4. The roughness data showed no normal distribution of the 121 Ra profiles in any subgroup. Thus, distributions were compared with Wilcoxon signed rank test.

**Figure 5:**
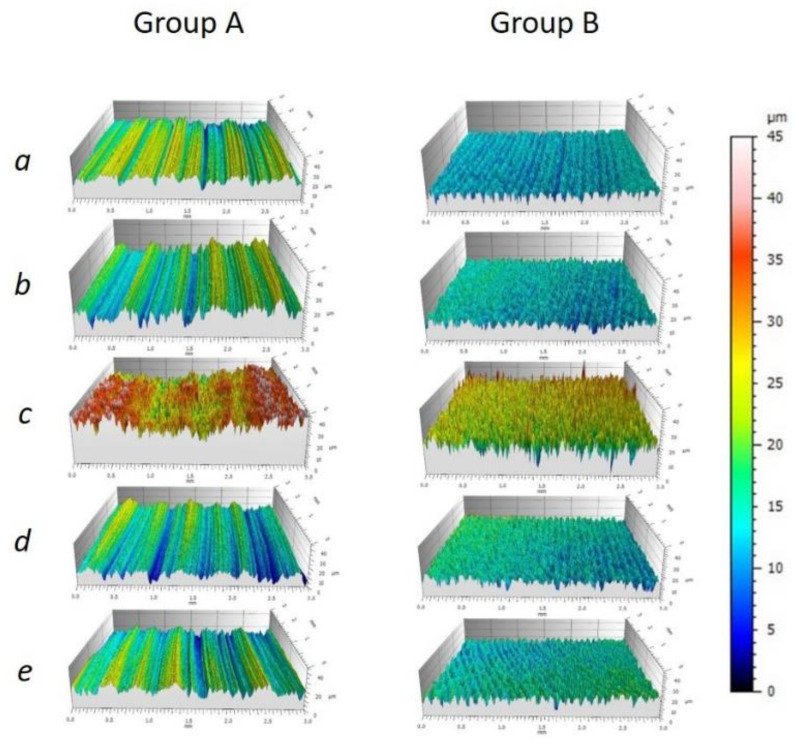
Exemplarily surface roughness profiles for Groups A and B showing the reference (**a**), MMA (**b**), sandblasted (**c**), oxygen plasma (**d**), and nitrogen plasma (**e**).

**Figure 6:**
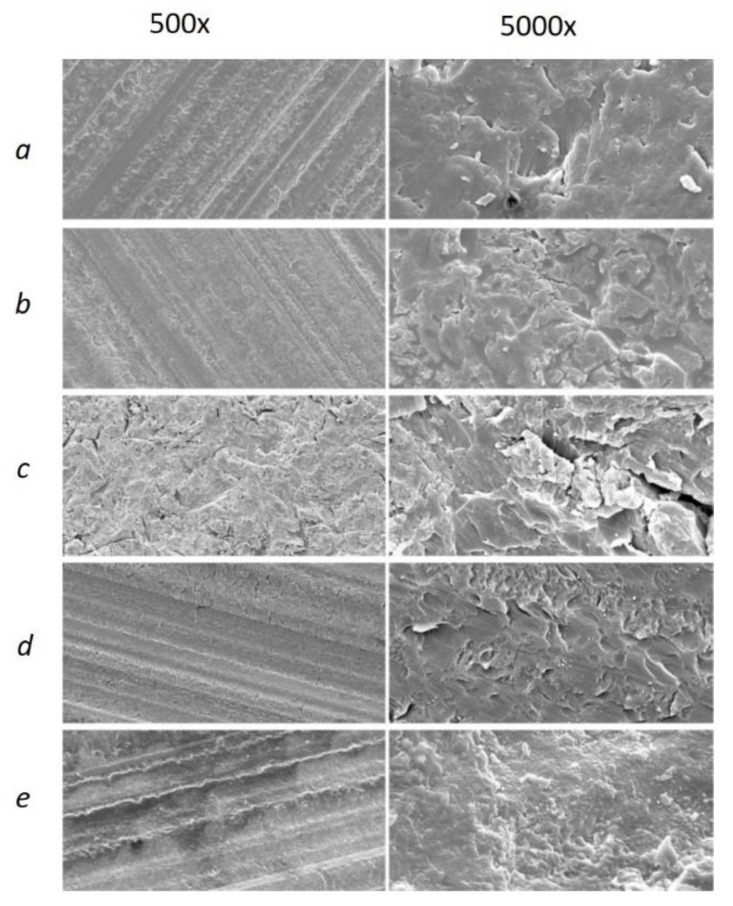
SEM images of Group A with 500× and 5000× magnification of reference (**a**), MMA (**b**), sandblasted (**c**), oxygen plasma (**d**), and nitrogen plasma (**e**) groups.

**Figure 7:**
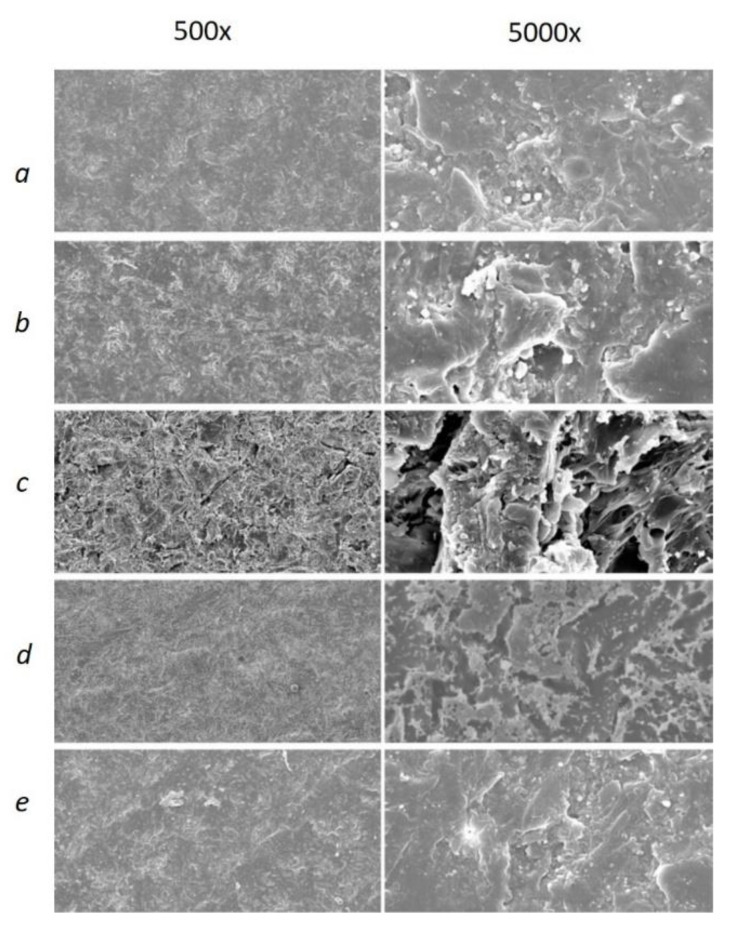
SEM images of Group B with 500× and 5000× magnification of reference (**a**), MMA (**b**), sandblasted (**c**), oxygen plasma (**d**), and nitrogen plasma (**e**) groups.

Figure 5 demonstrates the difference in specimen profiles after the several surface treatments. The specimens in Group A exhibited a stepwise lines profile for all groups except the sandblasting, whereas, in the Group B, the uniformly rough surface can be observed for all specimens. In both groups, the highest Ra values were found for sandblasting. The SEM images provided further visual evidence of different surface structures for both groups (see Figure 6 and Figure 7). Similar to what is depicted in the 3D profiles (Figure 6), the line-wise structure in Group A and uniform surface in Group B were observed in both 500× and 5000× SEM magnification, too (Figure 6 and Figure 7).

### 3.2. Shear Bond Strength

**Figure 8:**
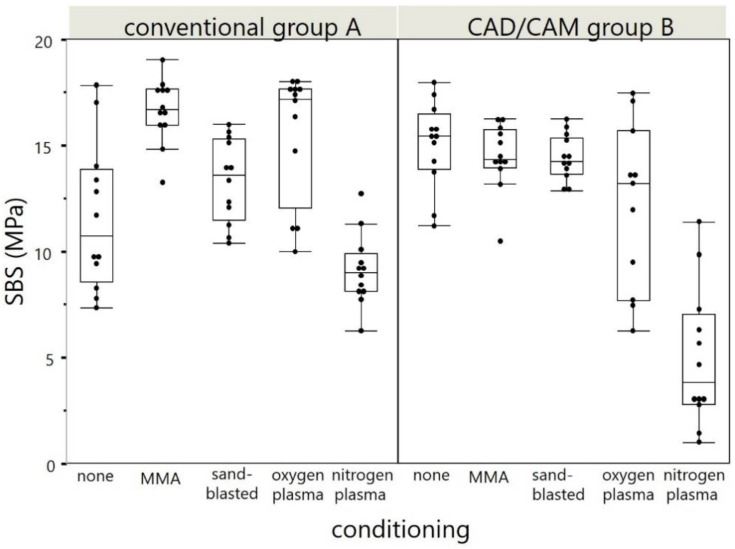
Boxplots of the shear bond strength (SBS) values for the Groups A and B. The data are derived from *n* = 12 specimens per mechanical test depending on each conditioning (*x*-axis).

The means and standard deviations of the calculated bond strengths are given in Table 2 and displayed in Figure 8. All distributions were normally distributed expect of nitrogen plasma in Group A (*p* = 0.004). In Group A, statistically significant differences were detected between the reference (none) and each subgroup with surface treatment. In Group B only the shear bond strength values of nitrogen plasma showed statistically difference compared to the reference group. No statistically significant correlation was observed between the roughness Ra (x-) and SBS (y-) values (−0.183; *p* = 0.083).

### 3.3. Modes of Fracture

**Figure 9:**
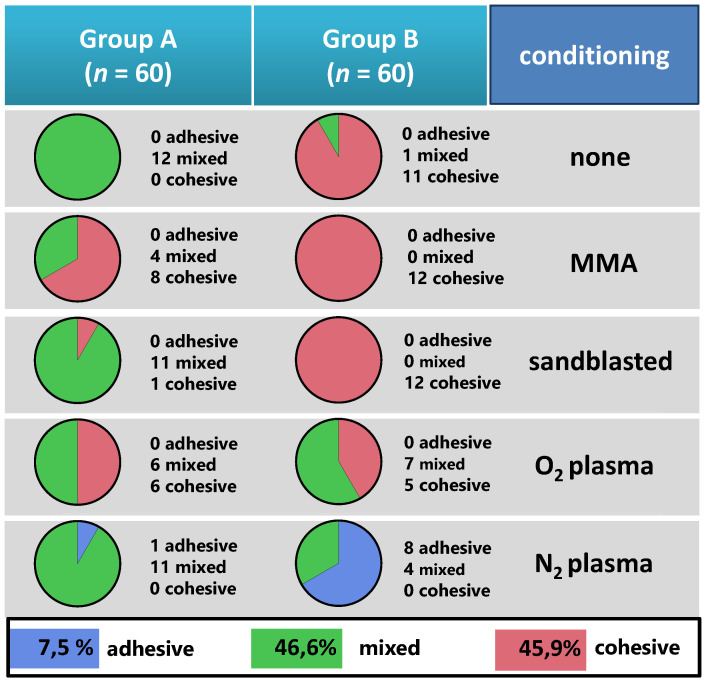
Fracture modes encountered after the shear bond testing. Pie charts with total numbers of adhesive (top), mixed (middle), and cohesive (below) fracture modes in the two experimental groups (Groups A and B) and five subgroups (surface conditioning).

Nine complete adhesive fractures were found in the nitrogen plasma groups (Figure 9). The mean fracture strength for adhesive fractures was 3.56 MPa (SD = 0.88).

The complete cohesive fractures (*n* = 54) had a mean fracture strength of 15.39 (SD = 0.36) and were found preferably in CAD/CAM base material (Group B) when no plasma was applied. The remaining 56 mixed fractures had a mean fracture strength of 11.62 (SD = 0.35).

Ultimately, 100% cohesive fractures were found only in the Group B. The analysis of 56 mixed fractures towards their mean share of cohesive fracture areas is shown in Figure 9. Herein, the cohesive fractures were found within a tooth-sided area in the conventional material modified with monomer (85%), followed by sandblasted surface (34%), no treatment/ reference (27%), oxygen plasma (18%), and nitrogen plasma (2%), as compared to the CAD/CAM material modified with oxygen plasma (13%) and nitrogen plasma (4%).

## 4. Discussion

Since statistically significant differences were revealed between the surface-treatment groups, the null hypotheses of the study have to be rejected. Though, clinically relevant differences were also detected since nitrogen plasma treatment revealed a shear bond strength below 5 MPa in the group with industrial polymerized PMMA after artificial aging.

The results are based on an investigation on the bond strength between denture teeth and bases, using a central upper incisor in 135° angulation [14,19]. The angle between the sample and the inspection stamp was chosen to be 135°, because, in clinical conditions, an artificial anterior resin tooth has an interincisal angle of 135° to the antagonistic teeth. This clinical adaption was found to be relevant in other investigations [24,25] and conveyed to the method.

According to the study of Zuckerman et al., the anterior upper incisors are mostly affected by debonding [24]. This seems to be comprehensible because molars and premolars have a larger bonding surface than incisors and do not experience a leverage of force transmission as incisors do while biting off. Therewith, molars are well supported by antagonists, and potentially smaller shear forces affect the posterior dentition during the mastication process.

In the topical literature, various approaches have been published to test the bonding behavior of prefabricated teeth to PMMA: some studies concentrated on the shear bond strength (SBS) testing [15,16,26], while other studies utilized the tensile bond strength (TBS) [26]. Both of these test types enable a good standardization, but they do not comprehensively simulate the clinical situation [27]. Forces applied to the central incisor during biting and mastication processes are rather a mixture of shear, tensile, and rotational stress. Therewith, the crosshead speed seems to have an influence on the shear bond strength. Literature reports speed values ranging between 0.5 and 2.0 mm/min [28]. However, an ISO guideline (TR 11405) recommends applying a crosshead speed between 0.45 and 1.05 mm/min (International Organization for Standardization, ISO TR 11405: Guidance on testing of adhesion in tooth structure, 1994). In the present study, the test setup was selected in accordance with existing ISO standards. Deviations or adaptions of the experimental conditions were carried out with regard to DIN 13998 (predecessor of TR 11405).

Prior to testing, all samples were thermocycled with 5000 cycles and 5 to 55 °C temperature change in water, in accordance with Palitsch et al. [27]. Thermocycling is also a deviation from ISO 13998, which does not include any procedure of artificial aging. This method allows us to imitate intraoral conditions, such as the influence of fluids and thermal variation on the dental prostheses, especially the bonding interface. However, with limitations of this laboratory simulation, it has to be stressed that this is only an abstract imitation of real chemical, thermal, and especially physical conditions, which daily influence the prostheses in the oral cavity.

With regard to the specimen fabrication and its transferability to real lab conditions, the contaminations of corresponding surfaces are the most reason for later debonding. Wax residuals especially impair the bond strength of a tooth to the denture base [16,17]. The production of the specimens with duplicate molds prevents such a contamination of the surfaces due to the absence of wax residues or alginate based insulating agents. Albeit favorable in the experimental setting, on the one hand, this impedes our ability to convey the data to daily practice in the dental lab, where contamination is much likely for conventional workflows. On the other hand, for digital workflows of CAD/CAM-based fabrication, the surface contamination with wax and other adherents is very unlikely.

The application of MMA yielded the highest SBS values between artificial teeth and PMMA. This finding coincides with the outcomes of Palitsch 2012 et al., who states that methylmethacrylat seems to be the golden standard and may increase the bond strength compared to other surface modifications [27]. Sandblasting also yielded a slight bond strength elevation. This was also an observation in the study of Mahadevan et al. after sandblasting of the surface for 30 s with 0.3 bar and 250 µm aluminum oxide [29]. However, the chemical solvent exceeds physical properties of the bonding interface.

Application of oxygen plasma yielded a slight bond strength elevation, which coincides with the outcomes of Aljudy et al. [30]. The utilization of nitrogen plasma yielded even the bond-strength reduction between both artificial teeth to PMMA and PMMA to milled denture base and therefore cannot be considered as a valid surface-treatment option.

The present study investigated the bonding behavior between milled bases from only one industrial polymerized PMMA with a manual mixed PMMA layer from powder and MMA monomer. With regard to the findings of Engler et al., it can be assumed that industrialized PMMA is differing in long-term bonding behavior when resulting in different monomer releases over time [10]. This should be taken into account for further studies, as well as the fact that, today, the production of denture bases tends to be performed in an additive way, using direct light processing and stereolithography methods. This indicates a major limitation of the present study that the integration of additively produced denture bases was not considered. This should be addressed in the future research. Furthermore, the current dental CAD software enables an individual design of denture teeth, which can be afterwards fabricated either in subtractive or additive way. Thus, further research should consider including various variations of subtractive and additive manufacturing methods of both denture teeth and bases. The further limitation of the present study is the fact that no sample size calculation was performed and the convenience sample size given by the current standard (DIN ISO/TS 19736:2018-09) was chosen.

## 5. Conclusions

Within the limitations of the present study, the MMA monomer application can be proposed as the most effective and efficient surface-treatment method for the integration of custom artificial resin teeth into a denture base milled from a PMMA CAM blank. Sandblasting and oxygen plasma application may also be considered as a reliable pretreatment option, but seem not to be efficient. Sandblasting yielded the greatest roughness values; however, no positive correlation on the SBS values was found.

Nitrogen plasma application is not recommended, as it may decrease the bonding strength between artificial teeth and PMMA even below 5 MPa.

## Figures and Tables

**Table 1:** Ra values (µm) of the different surface modifications. Data are based on *n* = 1 per group derived from 121 profiles every 25 µm over an area of 9 mm^2^ in the center of the specimen.

Subgroup	Group A (Resin Tooth) Ra		Group B (PMMA Base) Ra	
	Mean	SD	Mean	SD
none	2.17	0.05	1.46	0.17
MMA	2.09	0.08	1.55	0.18
sandblasted	2.31	0.3	1.96	0.3
oxygen plasma	2.1	0.08	1.6	0.17
nitrogen plasma	2.13	0.06	2.01	0.17

**Table 2:** Calculated bond strength in MPa as mean and standard deviation (SD) followed by pairwise comparison between the surface treatments within the groups (and statistical comparison between the two groups of materials (Groups A and B) for each surface treatment.

Subgroup Surface Treatment	Group A			Group B		
	SBS (MPa)					
	Mean	SD	Sign.	Mean	SD	Sign. *
none	11.59	3.49	AD	15.04	2.05	A
MMA	16.62	1.52	B	14.46	1.59	A
sandblasted	13.33	1.99	CD	14.44	1.07	A
oxygen plasma	15.53	3.04	BC	12.14	3.92	A
nitrogen plasma	9.12	1.69	A	4.93	3.28	B

* Corresponding letters indicate no statistical difference.

## Data Availability

All study data are available upon reasonable request.

## Supplementary Materials

The following are available online at https://www.mdpi.com/article/10.3390/ma14143822/s1, Supplementary File S1: S1_STL-File_Cylinder25x20.stl.

## Appendix A

**Table A1:** Description of Materials used in the study.

Brand Name	Company, City, Country	Composition Given by Manufacturer
Mondial 6 R483	Kulzer, Hanau, Germany	NanoPearls^®^-Material, PMMA based
megaCRYL N (liquid & powder)	Megadental, Büdingen, Germany	Powder: PMMA-Copolymer, dibenzoylperoxide, phenylbenzylbarbiturateacid, butyl-barbiturateacid, titanium dioxide, ferric oxide, peri-dinaphthylen. Liquid: MMA: butandioldimethacrylate, copper naphtenic acid, dilauryldimethylammoniumchloride, hydrochinone
Pink Blank U	Anaxdent, Stuttgart, Germany	polymethyl methacrylate, pigments
Korox 250	Bego, Bremen, Germany	250 µm Al_2_O_3_
Dublisil 30	Dreve Dentamid, Unna, Germany	addition-vulcanizing polyvinylsiloxane
